# Actions for stakeholders to develop better real-world evidence for HTA bodies/payers decision making

**DOI:** 10.1017/S0266462325100238

**Published:** 2025-06-17

**Authors:** Ashley A. Jaksa, Alina N. Pavel, Matti Aapro, Niklas Hedberg, Victoria Hodgkinson, Laurie J. Lambert, Francois Meyer, Matias Olsen, Piia K. Rannanheimo, Karen M. Facey

**Affiliations:** 1 Aetion, Inc., Boston, MA, USA; 2 RWE4Decisions Secretariat, FIPRA, Brussels, Belgium; 3 Genolier Cancer Center, Genolier, Switzerland; 4 Tandvårds- och läkemedelsförmånsverket, TLV (the Dental and Pharmaceuticals Benefits Agency), Stockholm, Sweden; 5 Lumiio Inc., Calgary, AB, Canada; 6 Department of Clinical Neurosciences, Hotchkiss Brain Institute, University of Calgary, Calgary, AB, Canada; 7 Canada’s Drug Agency, Ottawa, ON, Canada; 8 Independent Consultant, France; 9 European Confederation of Pharmaceutical Entrepreneurs (EUCOPE), Brussels, Belgium; 10 Finnish Medicines Agency, Fimea, Kuopio, Finland; 11 Usher Institute, University of Edinburgh, Edinburgh, UK

**Keywords:** real-world data, real-world evidence, decision making, stakeholder participation

## Abstract

**Objective:**

In 2020, RWE4Decisions, a multi-stakeholder initiative commissioned by the Belgian payer, published stakeholder actions to support the generation, analysis, and interpretation of real-world evidence (RWE) to inform the decision making of health technology assessment (HTA) bodies/payers for highly innovative medicines in the European Union (EU). Since 2020, changes in the decision-making environment and advancements in RWE have created an impetus to update stakeholder actions for the EU and Canada.

**Methods:**

RWE4Decisions’ experts led focus groups with individual stakeholder groups (HTA bodies/payers, pharmaceutical industry, clinicians, patients, registry holders, and data analytical experts). Each focus group crafted new actions for their stakeholder, then the actions were discussed and revised in a multi-stakeholder meeting, a public webinar, and a public consultation. Themes across actions and meetings were identified.

**Results:**

Detailed new actions for each stakeholder group are presented. Key themes identified are the need to address interorganizational fragmentation regarding secondary data use and methodologies to build robust RWE. HTA bodies/payers need to develop a common vision about the potential use of RWE. The role of the whole clinical team as primary data collectors is critical. Opportunities for scientific advice across the life cycle of a medicine are essential, and the implementation of RWE guidance related to HTA is paramount. Progress requires specific, operational actions and a collective effort by a variety of stakeholders.

**Conclusions:**

Carrying out these actions will facilitate the development of methodological best practices for generating RWE to inform HTA of highly innovative medicines and build trust between stakeholders in the use of RWE.

## Background

In 2020, RWE4Decisions, a multi-stakeholder initiative commissioned by the Belgian payer (the National Institute for Health and Disability Insurance [NIHDI]) (Box 1), published stakeholder actions to support the generation, analysis, and interpretation of real-world evidence (RWE) to inform the decision making of health technology assessment (HTA) bodies/payers for highly innovative technologies in the European Union (EU) (1). NIHDI has been hosting EU multi-stakeholder roundtables for several years regarding the generation of RWE over the life cycle of medicines. In 2019, a key conclusion was that other stakeholders, beyond the pharmaceutical industry (i.e., clinicians, patient groups, registry holders, analytic/academic groups, and regulators) had a role in generating robust RWE for decision making. This conclusion led to the development of the 2020 stakeholder actions using a multi-methods approach (1). When the actions were published, RWE4Decisions received feedback from Canada’s HTA body, patient groups, and registry holders that the proposed actions were also relevant in the Canadian environment.

Box 1. Description of RWE4Decisions and its mission.

RWE4Decisions is a learning network commissioned by the Belgian National Institute for Health and Disability Insurance (INAMI-RIZIV). The network comprises policy makers, HTA bodies, payers, regulators, clinicians, patient group representatives, researchers, data custodians (e.g., registry holders), data analytics, industry, and academic experts. Our work is driven by the core principles of **collaboration** and **transparency**:

***Collaboration***

- RWE generation is a shared responsibility and should be planned with all stakeholders and other decision makers, such as regulators.
- Iterative multi-stakeholder dialogues should be encouraged during the life cycle of a highly innovative medicine to discuss plans for evidence generation and the potential for RWE to resolve important decision-relevant uncertainties and plan for RWD collection.
- Each stakeholder needs to take responsibility for aspects they can influence and work collaboratively with other stakeholders to achieve the common goal of developing RWE that can inform payer/HTA decisions and improve patient care.

***Transparency***

Plans for RWD collection and generation of RWE should be shared publicly (in line with confidentiality agreements) to ensure that data sources can be designed, coordinated, and combined by:

- Clarifying what questions RWD may be able to address in payer/HTA decision making.
- Publishing case studies showing how RWE has been assessed in HTA.
- Sharing information about PLEG studies being planned across different jurisdictions to enable data aggregation.

RWE4Decisions’ mission is to bring together experts from all stakeholder groups to engage in dialogues that consider how fit-for-purpose real-world evidence (RWE) can be generated over the life cycle of highly innovative medicines by:

- Identifying medicines for which RWE generation would be the most useful.
- Identifying what RWE is needed to inform HTA/payers’ decisions.
- Advising on RWE generation plans.
- Clarifying how RWE will be assessed and used.
- Supporting planning and execution of effective post-launch evidence generation.

RWE4Decisions is governed by a Steering Group that includes at least one representative from each stakeholder group (citation). The Steering Group develops the priorities and agendas for roundtable discussions, workshops, and publications/outputs (citation). The goal of these activities is to provide actionable examples to facilitate learning for each stakeholder group.

Since the publication of these stakeholder actions, there have been changes in both the HTA and decision-making environment and advancements in the use of RWE for decision making in the EU. For example, in the EU, the Regulation (EU) 2021/2282 on Health Technology Assessment (2), mandated that from January 2025, Joint Clinical Assessments (JCAs) will be developed for all new oncology medicines and advanced therapeutic medicinal products and will be extended to all new orphan medicinal products in 2028. JCAs will deliver an assessment of the relative effectiveness of these medicines. The EU Member States shall pay “due consideration” to the assessment report in the national processes for HTA, pricing, and reimbursement. However, a more detailed understanding of “due consideration” needs further development, especially bearing in mind that the national processes, including evaluation of cost-effectiveness, remain the responsibility of the Member States, so fragmentation in the use of RWE is likely to persist.

Outside the EU, similar structural changes are impacting payers’ decision making. NHS England’s Innovative Medicines Fund enables interim reimbursement with Post-Launch Evidence Generation (PLEG) where it is determined that significant uncertainties can be resolved by real-world data (RWD) collection informing future reappraisal by the National Institute for Health and Care Excellence (NICE) (3). Canadian stakeholders have worked collaboratively to mobilize RWD sources (4) and the creation of Canada’s Drug Agency expands the Canadian Agency for Drugs and Technologies in Health’s (CADTH) remit (5) to increase RWD collection and analysis. International changes include an increase in highly innovative medicines receiving regulatory approval through accelerated approval processes, which can lead to less clinical evidence being available at the time of approval (6;7) and make HTA/payers’ decision making challenging.

In addition to changes in the HTA and decision-making environment, substantial progress has been made to advance the role of RWD in decision making. Health systems have increased the digitization of healthcare encounters, providing more opportunities for secondary use of data to inform decision making (8-11). This proved crucial during the coronavirus disease 2019 pandemic when fast access to relevant and reliable RWD was needed to inform optimal treatments and vaccination strategies. Similarly, the adoption of common data models, which standardize the structure and format of data across different sources, is becoming more commonplace. At the national level, Spain (12) and Italy (13) have developed bespoke monitoring registries to collect RWD post-launch to address uncertainties and inform pricing and reimbursement renegotiations, whereas countries like Finland, Norway, Canada, and Scotland are recognized as having good health data sources and abilities to link data via a unique citizen identifier (4;14).

Cross-border cooperation in collecting and analyzing secondary use data is challenged by legal, organizational, semantic, and technical interoperability issues. However, in the EU, the European Health Data Space Regulation (EHDS) (15) aims to facilitate secure exchange and reuse of health data, and the General Data Protection Regulation (GDPR) sets the general legal framework for collecting, storing, and managing personal data. GDPR’s consequences include obtaining a legal basis when using RWD, such as public interest or explicit consent, and the need for data minimization and robust pseudonymization measures. It also imposes transparency obligations, increases administrative burden through, for example, Data Protection Impact Assessment, and restricts cross-border data transfers. In addition, the Data Governance Act and Data Act promote data sharing and fair access to data, and the Network and Information Systems Directive enhances cybersecurity for health data (15).

Stakeholders have also advanced RWE science to meet the needs of decision makers. The European Medicines Agency has made major strides in data analytics (16) including the development of Data Analysis and Real-World Interrogation Network (17) – a federated network that addresses RWE questions relevant to regulators and HTA/payers. Collaborative demonstration projects have improved analytical methods (18-20), while initiatives like RWE4Decisions facilitate dialogue on building robust RWE for HTA/payers (1) and collecting RWD to resolve uncertainties through PLEG (21). Pharmaceutical companies now consider RWE generation over the life cycle of a medicine, and analytics experts are developing methodologies to validate RWE and support transparency. These advancements have informed regulatory and national HTA bodies’ guidance, which articulates standards for RWE submissions and helps improve the usefulness, quality, and reporting of RWE studies (e.g., see (22-25)).

While the diligent and collaborative work by all stakeholders, including those involved with RWE4Decisions, has addressed many of the 2020 recommended stakeholder actions, substantial challenges remain in optimizing RWE generation throughout a medicine’s life cycle and ensuring its suitability for all decision makers. Multiple articles (e.g., see (26;27)) have outlined the current challenges to incorporating RWE studies in HTA and payers’ decision making. These challenges typically fall into four pillars (27): data (e.g., availability of local high-quality RWD), methodology (e.g., explanation of RWD selection choices, bias adjustments, and appropriate methods), trust (e.g., lack of transparency), and policy and partnerships (e.g., harmonized guidance). As each of these types of challenges is relevant to multiple stakeholder groups, a continued collaborative effort that considers the changes to the decision making environment is needed. In response, RWE4Decisions has reviewed and updated the 2020 stakeholder actions to support the use of RWE in HTA/payers’ decision making for highly innovative medicines in Europe and Canada, involving a wide range of stakeholders in closed and open meetings.

## Methods

The 2025 stakeholder actions were developed through stakeholder focus groups, multidisciplinary roundtable discussions, a public webinar, and written public consultation, all conducted in English. Stakeholder groupings from the 2020 paper were retained, except for regulators. Stakeholders agreed that regulators are developing strong RWE assessment and generation strategies, as shown in EU and international publications, so they were excluded. Payers and HTA bodies were combined in the 2020 actions and retained in the 2025 update. This is because the roles and responsibilities of organizations vary across Europe, with HTA bodies assessing RWE in some countries, while the payer does so in countries without an HTA body, so differentiating actions into separate groups is challenging.

Virtual focus groups were undertaken in January 2024 with eight stakeholder groups: national HTA/payers (nine participants), collaborative HTA/payer’s initiatives (five participants), pharmaceutical industry (ten participants), healthcare providers (six participants), rare disease patient advocates (four participants), cancer patient advocates (four participants), registry holders (six participants), and data analytics experts (nine participants). Participants in all stakeholder groups were from Europe and Canada; the data analytics group included experts from the United States, as RWE methodology is country-agnostic. Each focus group was co-moderated by two of the authors with relevant expertise. Discussions covered recent and upcoming policy or system changes affecting RWD collection, a review of the 2020 actions identifying the successes and challenges in implementation, and the development of new actions, which were later refined by email.

The first draft of the 2025 Stakeholder Actions was discussed in February 2024 in a multidisciplinary virtual roundtable including forty-nine participants from the RWE4Decisions Learning Network (sixteen HTA/payers, fourteen industries, four patient representatives, five analytics experts, three registry holders, five academics, one EU institution representative, and one international organization representative). Participants, senior representatives from their organizations, were predominantly from the EU, with at least one representative from the United Kingdom or Canada in most groups. Author-moderated breakout groups discussed key actions, gaps, and potential implementation challenges. After the meeting, all participants were encouraged to comment on all stakeholder groups’ actions. A second draft of the actions was prepared and approved by the authors.

RWE4Decisions held a public webinar in March 2024 to present the second draft of actions (28), using polling to gather feedback on the priority actions for the HTA/payers and industry stakeholders. Further revisions followed. RWE4Decisions requested feedback from organizations during a written public consultation in June–July 2024, which drew eleven responses (four submissions from industry, two from academics, two from patient groups, two from HTA bodies, and one clinician). Following the written consultation, the actions were categorized into the most relevant of the four pillars: data availability and governance, methodology, trust and transparency, and policy and partnerships (27). Final revisions were made by co-authors in August 2024, incorporating all feedback.

The focus groups and multidisciplinary roundtable discussions operated under the Chatham House Rule to enable frank discussion. For manuscript development, two authors (AJ and KF) reviewed meeting documentation, written public consultation feedback, and the final stakeholder actions, identifying themes later discussed and refined with coauthors; themes emerged in the discussions that shaped the actions, and themes emerged in the actions themselves.

## Results

### Themes identified from the focus groups and roundtable discussions

During the stakeholder focus groups and roundtable discussions, all stakeholder groups noted how the landscape for RWE generation and use has changed substantially since 2020, prompting substantial changes to the list of actions. When discussing how to update the actions, common themes across stakeholder groups emerged, which guided the development of the 2025 Actions (Table 1). The themes were as follows:

- Continued **interorganizational fragmentation** is a barrier to progress in the use of RWD/E in decision making and a major concern.
  For example, pharmaceutical industry representatives highlighted the lack of consensus among HTA bodies/payers on acceptable RWE methodologies and their use in submissions, making it difficult to meet diverse requirements. Clinicians raised concerns about data silos caused by strict privacy laws (i.e., the inability to access a patient’s data from a different clinic). Payers noted challenges in accessing and analyzing medication use information due to data privacy rules.
- A **common vision** is needed among payers/HTA bodies regarding the use of RWD and the evaluation of RWE.
  Some HTA bodies are advanced in articulating RWD/RWE requirements (24), while others are still developing their expertise. Some health systems have a robust infrastructure that facilitates comprehensive data capture and analysis, while others face challenges in assessing and utilizing healthcare data. Greater clarity on what constitutes “good” RWD/E would benefit multiple stakeholders. Based on the stakeholder discussions, the standardization of all HTA RWE guidance is not feasible due to different health system structures, legal mandates, and assessment and appraisal approaches. However, harmonization would be helpful. This requires strong leadership to create and share a common vision among payers and HTA bodies and collaboration with regulators, especially given the advancements made by regulators in the EU.
  All stakeholders felt they would benefit from this common vision. For example, it could inform registry holders and clinical teams about key data to collect and how to ensure its quality. Clarification of the payer’s/HTA’s vision could inform the pillars that seek to build RWE (27) by developing consensus on data ecosystems to collect robust RWD (data pillar), harmonizing RWE methodological standards (methodology pillar), and even harmonizing policies between HTA and payers within a single country (policy and partnerships pillar).
- **Continue the development and establishment of RWE guidance.**
  Remarkable progress has been made in RWE guidance, with 50 guidance documents published from 29 organizations since 2017 (29). Notably, NICE released its RWE Framework in 2022 (24), outlining when RWD can address uncertainties and describing best practices for the design, conduct, and reporting of RWE studies. In 2023, CADTH guidance focused on reporting RWE studies (25) aligning with existing international guidance. Critical evaluations of the guidance documents have noted that RWE best practices are emerging, but that gaps remain (e.g., in assessing “fitness for use”) and harmonization is needed (29-31). Focus group and roundtable participants agreed that a more concerted and collaborative effort at guidance harmonization is necessary and that additional guidance should not be duplicative.
- In the 2020 actions, **collaboration across stakeholder groups** was essential and is a continued theme in the 2025 actions.
  For many actions, multiple stakeholder groups should be involved and share responsibilities; the onus should not just fall on HTA bodies and/or industry.
- Actions should be **more specific and operational**.
  For example, “*Encourage industry to engage in multi-stakeholder dialogues to discuss evidence generation plans including RWD collection*” (Action 1.2, 2020) was updated to “*Collaborate to expand early dialogue/scientific advice/Joint Scientific Consultation to cover RWE generation throughout the product life cycle to address uncertainties in clinical and cost effectiveness. Recognise the need for agile processes to adapt to evolving knowledge, treatment options and data and evidence requirements*” (Action 2.5, 2025; for a direct comparison of the 2020 and 2025 actions see Supplemental Materials). Stakeholders agreed that more specific actions are easier to operationalize and facilitate measurement of success.
- **Scientific advice continuing across the medicine’s life cycle and iterative RWE planning is essential.**
  One emerging theme was the importance of planning RWE across the life cycle of a medicine, starting early and involving multiple stakeholders. Such discussions in scientific advice processes (e.g., EU Joint Scientific Consultations (32) or Canada’s Drug Agency Scientific Advice program (33)) can outline gaps in clinical development plans that could be filled with robust RWE. Stakeholders could agree on priorities and discuss the feasibility of RWD collection.
- Recognizing that **entire clinical teams are often responsible for collecting RWD** rather than clinicians alone.
  The 2020 actions referred to “clinicians,” but in roundtable discussions, the importance of the whole clinical team for capturing and recording health and administrative data was emphasized and, at a higher policy level, the term “clinical networks” has been used (e.g., EU cancer networks).

**Table 1.** Stakeholder actions to generate better RWE for HTA/payer decisions

**1. Payers and HTA bodies: national payers/HTA bodies**		
*Policy and partnerships*		
	Create a shared vision that conceptualizes a future state for the use of real-world data (RWD) in health technology assessment (HTA)/payer processes. Ensure that clear roles and responsibilities are designated for oversight and achievement of the shared vision.	
	Overcome fragmentation and lack of collaboration between HTA bodies and payers by implementing the necessary infrastructures, aligning processes, and upskilling competencies for effectively requesting, producing, and utilizing real-world evidence (RWE).	
*Data availability and governance*		
	Collaborate with other HTA bodies and payers (e.g., via networks such as the National Competent Authorities for Pricing and Reimbursement (NCAPR) or the Medicine Evaluation Committee (MEDEV)) and regulatory authorities to align post-launch evidence generation (PLEG) requirements needing national data collection. Focus on critical endpoints and requirements for data quality.	
	Initiate and engage in multistakeholder discussions with companies about RWE’s generation plans. Determine which data are transferable and identify additional local RWD that may be needed to inform decision making, aligning with other jurisdictions when possible.	
	Influence national developments on the secondary use of health data. Communicate HTA/payer needs regarding, for example, types of data, data linkage, and data quality, ensuring these needs are considered and integrated into national governance frameworks.	
	Require that feasibility assessments, study protocols, details of data extractions, and study reports are made publicly available.	
	Publish examples where RWE has influenced pricing and reimbursement decisions or reassessments. Also, share case studies that identify methodological areas requiring development.	
**2. Payers and HTA bodies: payer/HTA collaborations**		
*Policy and partnerships*		
	Use HTA/payer collaboratives (such as Joint Nordic HTA-Bodies, the Beneluxa initiative, etc.) to encourage and enhance joint work on use of RWD in initial access decisions, managed entry agreements, and reassessments.	
	Develop a joint voice on RWD issues to feed into policy and system developments, such as the European Health Data Space (EHDS) and the Data Analysis and Real-World Interrogation Network (DARWIN-EU), and the implementation of the HTA Regulation, with clear accountability for this task potentially assigned to an entity such as NCAPR.	
	Co-create joint learning platforms with other decision makers and stakeholders in the RWD/E community.	
	Work with regulators to understand and influence their international activities to develop harmonized methods and guidance for RWD collection. In Europe, incorporate RWD/E needs and guidance in the implementation of the HTA Regulation through Joint Scientific Consultations and Joint Clinical Assessments (JCAs).	
	Collaborate to expand Early Dialogue/scientific advice/Joint Scientific Consultation to cover RWE generation throughout the product life cycle to address uncertainties in clinical and cost-effectiveness. Recognize the need for agile processes to adapt to evolving knowledge, treatment options, and RWD and RWE requirements.	
	Encourage and support industry to consider possibilities to expand post-authorization studies and data collection plans intended to support regulatory decision making, to also address HTA/payers’ evidence needs	
	Collaborate with companies, clinical teams, academia, and other stakeholders on study protocols, study governance, analyses, and reporting to encourage a common understanding of HTA requirements and to promote open access to documents and findings.	
*Data availability and governance*		
	Collaborate with regulators on common frameworks for data quality assessment, data standardization efforts, and methodologies for feasibility assessment. Advise health data holders of the common requirements so that they can develop their datasets accordingly.	
*Methodology*		
	Pilot the use of existing RWD/E methods guidance and tools in assessments. Share feedback with guidance authors and the HTA/payers’ community.	
	After piloting existing guidance, develop harmonized RWE guidance jointly with academia and industry, particularly to support implementation of the HTA Regulation. Review the guidance regularly to take account of new methodological developments and experience of use (living guidance).	
*Trust and transparency*		
	Compile available documents (protocols and reports) describing examples of post-launch RWD collection and RWE generation required by HTA/payers, utilizing an existing portal if available.	
**3. Pharmaceutical industry**		
*Trust and transparency*		
	Discuss plans for RWE generation as part of industry (integrated) evidence plans at scientific advice meetings. Do this at several points during the life cycle of the medicine, to develop understanding of what RWE might be valuable to HTA/payers as clinical evidence and knowledge about the technology/disease evolves.	
	Ensure transparency around the design, conduct, and analysis of RWE studies that are agreed to be pivotal to HTA/payers’ decision making, for example, using published tools to document data capture, management, and analysis, following RWE guidance/frameworks.	
*Policy and partnerships*		
	Work together across company functions and national affiliates involved in RWE generation and analysis to develop a common understanding of national/regional HTA/payers’ needs for RWE.	
		Lead discussions about the transportability of RWD across borders and support efforts to align data collection requirements across jurisdictions.
	Continue to drive discussions about the use and alignment of Outcomes-Based Managed Entry Agreements (OBMEA)/PLEG.	
*Data availability and governance*		
	Explore the use and analysis of digital apps to capture patient-relevant outcomes, particularly to inform OBMEA.	
	Collaborate across companies (e.g., in a specific disease area or for a class of medicines) to agree on synergies in methods and processes for RWE in that specific case (e.g., core datasets, analytical approaches, etc.).	
	At an early stage of product development, engage with clinical networks to create or further develop registries and databases following unified data standards, to collect high-quality and interoperable data.	
*Methodology*		
	Engage and support operationalization of the HTA Regulation to highlight the need for RWE in the first two tranches of JCAs and encourage development of clear guidance about assessment of RWE in the EU HTA context.	
	Share, publish, and enable discussion of case studies to show how RWE has been assessed and used in HTA/payers’ decision making (in assessment and post-launch), focusing on specific issues (e.g., external control arms, transportability), challenging case scenarios (e.g., rare disease), and so forth.	
**4. Clinical teams**		
*Policy and partnerships*		
	EU clinical networks should engage in developments related to the European Health Data Space to support public and political awareness of the value of secondary use of health data.	
	EU clinical networks and clinical trials collaborative groups (such as European Reference Networks, the EU Cancer Mission Networks, the European Organisation for Research and Treatment of Cancer, and other disease-specific study groups) should systematically involve patients to collect patient-relevant outcomes, including nutritional status and comorbidities, and collaborate with regulators and payers/HTA bodies to ensure data collection systems are fit for all purposes, including HTA requirements pre- and post-launch.	
	Medical faculties should include educational programs on the value of health system data to improve the delivery of care, patient outcomes, and inform health system decision making, such as HTA evaluations of new healthcare interventions.	
*Data availability and governance*		
	Clinical networks and study groups should advise on the most suitable and efficient way for health systems to collect RWD to avoid multiplicity of data entry and clarify the support clinical teams require to collect good-quality RWD.	
	Clinical networks and clinical trials collaborative groups should encourage health systems to involve clinical teams and patients in the design of data collection systems and associated governance structures to ensure processes are efficient and clarify the support clinical teams require to collect good-quality RWD.	
	Clinicians should seek to ensure that local information governance systems are designed to deliver optimal care for patients (across providers and departments), and to encourage informed consent processes based on unified ethical principles that are intelligible to patients, in all required languages.	
**5. Patient groups**		
*Policy and partnerships*		
	Ensure that opportunities and resources to develop patient expertise in the field of RWD are clearly communicated to the patient community to develop skills that support multi-stakeholder and patient-centered generation of RWE to inform HTA and to engage in policy and system developments relating to the use of health data.	
	International patient groups should continue to engage in RWD initiatives, such as Innovative Health Initiative (IHI) projects, regulatory and HTA-led activities, and policy developments such as the EHDS.	
	Seek to influence the implementation of the EHDS and understand its implications for national data collection systems, in particular to ensure that patients have access to their own data.	
	Support the development of a process for iterative multi-stakeholder dialogues throughout the life cycle of a medicine to encourage alignment of views on identification, collection, analysis, and evaluation of RWD for decision making.	
*Data availability and governance*		
	Support the development of efficient informed consent processes for secondary use of health data. This will necessitate a rethinking of the type of consent model in use from “broad” to “dynamic” to encourage accelerated and increased patient participation.	
	Disseminate clear, unbiased, patient-relevant information about RWD and RWE to patient communities, including the value of secondary use of data and information to support PLEG.	
*Methodology*		
	Engage with clinicians, academics, and decision makers to discuss how patient-relevant data from novel collection methods (such as wearables, apps, etc.) can be used in decision making, to help build a predictable pathway for the use of these novel RWD collection approaches.	
	Patient groups who might also be disease registry holders or have developed disease-specific data collection approaches should agree with stakeholders on how to integrate such evidence with other data for highly innovative medicines.	
**6. Disease registry holders**		
*Policy and partnerships*		
	Participate in multi-stakeholder dialogues about RWE generation for a specific medicine, or group of medicines, to discuss the potential for a disease registry to be used for regulatory and HTA/payer purposes and ensure realism about data quality (availability for main outcomes and trade-offs for different data capture algorithms).	
	Form multi-stakeholder partnerships to support the use of data for HTA/payer purposes, including public/private partnerships.	
*Trust and transparency*		
	Engage with HTA/payers and industry to explain the construct and purposes of disease registries, discuss their potential and limitations, and agree with HTA/payers on how disease registries and RWE studies will be assessed, building on existing tools (e.g., tools to report registry quality overall and fitness-for-purpose evaluations for individual RWE studies).	
	Share and publish case studies of where RWD from disease registries has been used to support HTA/payers’ decision making.	
*Data availability and governance*		
	Develop governance processes that enable sharing of disease registry data and linkage to other data sources (e.g., administrative data) for HTA/payer purposes (including patient consent mechanisms, protocols, data management, etc.).	
	Align to RWD standards to ensure data meets the quality standards required by HTA bodies.	
**7. RWD/analytics groups**		
*Methodology*		
	Support the development of a repository of empirical evidence/case law about what RWE was fit for purpose in HTA and what was not, as well as RWE case studies that highlight data quality and methodology examples.	
	Continue to operationalize and share/publish methodologies that address known HTA/payers’ concerns with the use of RWE through HTA-friendly tools, demonstration projects, case studies, and so forth.	
	Build RWE analytics knowledge base and support RWE assessment and generation within HTA bodies, linking to new policy initiatives such as the HTA Regulation.	
	Work with HTA bodies to share and operationalize the implications from available demonstration projects (e.g., emulation studies, newly developed methods, tools, repositories, etc.) to build mutual understanding and trust in RWE; explore which are most helpful or need adaptation and support collaboration to create a harmonized RWE toolkit.	
	Raise awareness of differences between HTA body RWE frameworks and how these differences relate to differences in HTA body remits and RWE needs. Where appropriate, engage in dialogue with HTA bodies to encourage harmonization of fit-for-purpose methods.	
	Where there is substantial evidence and agreement across stakeholders on use cases, standards, and analytical methods, and where articulating guidance would benefit researchers, support HTA bodies in developing detailed, published guidance.	
	Create a standard worldwide library of definitions for key aspects covering diagnoses, outcomes, covariates, and so forth, and algorithms that have been validated.	
*Trust and transparency*		
	Work with industry to ensure they are following published standards/best practices for RWE generation (e.g., transparency, data quality, etc.).	
	Work with data custodians to explain the requirements of decision makers to clearly demonstrate data quality and encourage standardized documentation they can use for all RWE studies.	

### Feedback during the public webinar on updated actions

During the public webinar (28), the draft actions were well received, where polling identified priority actions for HTA bodies and industry. For HTA bodies, there was a clear agreement on the importance of draft action 1.2: “*Overcome the lack of collaboration and fragmentation between HTA bodies and payers by developing joint processes and upskilling competencies for requesting, producing, and utilizing RWE effectively. RWD collection for individual assessments should be aligned in terms of critical endpoints and data requirements for value assessment, decision-making, managed entry agreements, and regulatory needs*” (updated to final action 1.2, Table 1). For HTA/payer collaborative initiatives, the action deemed most important was draft action 2.3: “*Improve relationships with regulators to co-design RWD collection to suit the needs of regulators and payers*” (updated to final action 2.4, Table 1). For industry, the following actions were prioritized: draft action 3.1: “*Discuss plans for RWE generation as part of industry (integrated) evidence plans at scientific advice meetings. Do this at several points during the lifecycle of the medicine, to develop an understanding of what RWE may be valuable to HTA/Payers as clinical evidence and knowledge about the technology/disease evolves*” (updated to final action 3.1, Table 1) and draft action 3.2 “*Ensuring transparency around the design, conduct, and analysis of RWE studies*” (updated to final action 3.2, Table 1).

Public webinar feedback informed updates to the actions. For example, some roundtable participants suggested merging actions from national payers/HTA bodies and payer/HTA collaboratives, but polling revealed no consensus, so the actions were kept separate.

### Refinement and final version of 2025 actions

Written feedback from the public consultation led to minor edits, primarily to improve clarity.

## Discussion

Since the 2020 stakeholder actions were published, there have been substantial changes to the decision-making environment, RWD infrastructure, and RWE sciences. These changes provide more opportunities for secondary use of health data, promote methodological advancement in building and reporting, and highlight the potential value of robust RWE to resolve decision-relevant uncertainties. This progress has resulted from diligent work by all stakeholders, which was often aligned with the 2020 recommended stakeholder actions, such as improving the quality and accessibility of RWD, advancing analytical methods, improving transparency, collaborating on demonstration projects, and developing guidance for researchers generating RWE. However, this foundational work has not always translated to efficient and robust use of RWE in HTA, reimbursement, and managed entry processes. The 2025 actions seek to build on the new developments emerging in the health data ecosystem and HTA/payers’ decision-making processes to accelerate progress toward greater use of better RWE in HTA/payers’ decision making. We encourage widespread dissemination of these actions and translation into other languages.

A key challenge noted by stakeholders is the lack of harmonization between regulators and HTA bodies/payers and between decision makers in a particular jurisdiction. The 2025 actions promote a joint vision of RWE (i.e., what can be produced, by whom, and for what purpose), while recognizing the distinct settings in each country in terms of data infrastructure and decision making. For example, actions for HTA/payer collaborations call for the development of a shared voice on resolving RWD issues (e.g., data collection and access) that are relevant to reducing decision uncertainty but are GDPR compliant for both regulators and HTA/payers. Any actions on data ecosystem harmonization must consider recent legislation (e.g., Artificial Intelligence regulation) and learnings from legal challenges concerning the implementation of interoperability. Industry and national HTA bodies/payers emphasize the need for greater alignment on PLEG requirements and ideally, a core dataset focused on key decision-relevant uncertainties for HTA bodies/payers. Patients and clinical teams have a role to play by supporting the development of efficient and harmonized data collection systems and informed consent processes to facilitate research that addresses uncertainties (e.g., the Data Saves Lives initiative (34)). A more harmonized approach to collecting fit-for-purpose RWD around agreed-upon prioritized uncertainties would give the industry a clearer direction and improve evidence generation. Cross-jurisdictional collaboration to align post-launch data needs across regulators and HTA bodies/payers can increase efficiency and enhance patient access.

While multi-stakeholder collaborations are essential for progress, leadership roles and implementation timelines are often unclear. For example, one payer/HTA collaboration action (Action 2.3; Table 1) is to collaborate with regulators to align on data quality, standardization, and feasibility methods and guide data holders on meeting shared requirements. We did not specify whether regulators or payers/HTA bodies are ultimately responsible.

For all collaborative actions, the roles and responsibilities of different stakeholders will depend on the nature of collaborations already in place. In the EU, the HTA Coordinating Group leads the implementation of the HTA regulation. For example, Article 43 of the regulation indicates that the EU should continue to support voluntary cooperation on HTA between Member States and “facilitate synergies … with a view to providing additional RWE relevant for HTA (2).” However, national RWE is still important, but the role of the HTA Coordinating Group in supporting RWE collaboration is still undefined. Similarly, EU payers coordinate under the National Competent Authorities for Pricing and Reimbursement, but this body has not addressed issues relating to RWE. Globally, HTA collaboration is increasing; for instance, NICE is working with CDA and ICER in the Health Economics Methods Advisory group (https://icer.org/health-economics-methods-advisory-hema/). Given the varied mandates of these collaboratives, no single stakeholder may have the capacity or expertise to lead RWE efforts alone. Multi-stakeholder initiatives – like WHO Europe’s Novel Medicines Platform – may have a key role in advancing RWE and outcome-based agreements (https://www.who.int/europe/groups/the-novel-medicines-platform). A multi-stakeholder approach could provide collaboration across multiple stakeholders and could draw on funding from Horizon Europe or the Innovative Healthcare Initiative. While setting timelines to complete the actions would be ideal, progress depends on the infrastructure for collaboration across stakeholder groups and the pace of existing collaborative efforts.

RWE is only as good as the underlying data, yet collecting and accessing high-quality data remain a challenge. Although the EHDS will improve data governance in the EU, challenges in improving the quality of data captured in our health systems remain. Patient groups and clinical teams play a major role in developing efficient collection that supports optimal delivery of care. Often, clinical team members, whose primary role is not research, are responsible for entering information into registries, electronic health records, and claims databases. Improving data collection starts with a wider understanding of the value of healthcare data for many purposes and the potential for the use of artificial intelligence in recording and structuring data (35). Clinical Team actions focus on supporting “*public and political awareness of the value of secondary use of health data*” (Action 4.1, Table 1) and “*educational programmes on the value of health system data* to improve patient outcomes and the delivery of care” (Action 4.3). Patients have a particular role in helping to ensure “patient-relevant” data are collected (Action 5.7) and Clinical Teams should advise on the “*most suitable and efficient way*” to collect RWD (Action 4.4). Improving the quality of collected data will go a long way in improving the quality of and trust in RWE, and involving key stakeholders is essential to progress.

Analysis of RWD requires specific expertise beyond the typical skills of systematic review and economic modeling found in HTA bodies. Upskilling staff, sharing knowledge, and collaborating with academia and analytical experts are essential. For instance, RWD/Analytics Groups should “*build RWE analytics knowledge base and support RWE assessment and generation within HTA bodies*” (Action 7.3, Table 1) and “*collaborate with Payers/HTA bodies on developing harmonized RWE guidance*” (Action 2.10). Disease registry holders can support payers/HTA bodies by clarifying registry purposes and agreeing on how registries and RWE studies will be assessed (Action 6.3). These efforts will enhance trust in RWE studies and increase reviewer confidence in their methodology.

Transparency remains a challenge across stakeholders as each RWE study requires many decisions on study design and data handling. HTA bodies seek clearer reporting to verify preplanned decisions and avoid biased results (Action 1.6). Meanwhile, industry is concerned about the extent to which their commercially sensitive data, plans, and intellectual property could be accessed by competitors. Updated actions encourage industry to work with RWD/analytics groups to “*using published tools to document data capture, management and analysis*” (Action 3.2, Table 1) and “*ensure they are following published standards/best practices for RWE generation*” (Action 4.8). Additionally, greater collaboration across pharmaceutical companies to “*agree on synergies in methods and processes for RWE*” (Action 3.7) for a specific disease, especially rare diseases where information needs are high and knowledge evolves rapidly, would be beneficial to improve transparency and potentially address common uncertainties that arise during HTA. Payers/HTA bodies can also increase transparency by “*publishing examples where RWE has influenced*” (Action 1.7) decision making, like NICE has done in its RWE Framework, and publishing PLEG protocols, progress, and reports. Some agencies publish PLEG plans (such as Canada’s post-market drug evaluation program (36)) and Valtermed in Spain (12) publishes PLEG protocols, a dashboard of progress, and final reports. In other agencies, plans are embedded in pricing and reimbursement decision databases (e.g., AIFA in Italy (13)) and may be hard to find. The establishment of a public portal for such documents is proposed in Action 2.11.

The limitations of our work need to be acknowledged. First, while stakeholders who had a major role to play in RWE generation and use were included, there may be other stakeholders who were unintentionally excluded. Furthermore, all stakeholder engagement was conducted in English, which may have limited the participation of some experts. While the focus of the stakeholder actions is on European and Canadian stakeholders, we believe that some actions may be relevant in other jurisdictions, since there is interest in this topic in Australia, Asia, and South America. Finally, articulating the stakeholder actions does not guarantee that stakeholders will follow them or that they will make a substantial impact on the use of RWE in HTA/payers’ decision making. However, each stakeholder group was involved in the development of their actions and believed they were achievable. The correlation between several actions identified in the 2020 paper and the increased interest in the use and acceptance of RWE suggests that the successful completion of the 2025 actions will lead to increased interest and use of RWE for HTA/payers’ decision making for highly innovative medicines.

## Conclusions

Stakeholder groups are actively working to improve data collection and RWE methodology, and transparency. This is helping to improve understanding of how RWD/E can be used efficiently to inform and improve decision making. Ideally, stakeholders’ work will focus on those actions that will benefit the field most and ensure the efficient use of resources as we push forward the collection of high-quality healthcare data that can be used for optimizing individual patient care and a variety of secondary uses that will aid decision making and support health system sustainability. We have established actions for HTA bodies/payers, the pharmaceutical industry, clinical teams, patients, registry holders, and analytic groups. The actions are highly intertwined and will require multi-stakeholder collaboration. A more concerted and collaborative effort will build trust between stakeholders and in RWE and enable decision makers to clearly articulate their vision for the use of RWD/E.

## Supporting information

Jaksa et al. supplementary material Jaksa et al. supplementary material

## Supplementary material

The supplementary material for this article can be found at http://doi.org/10.1017/S0266462325100238.
