# Supplementary material for: Actions for stakeholders to develop better real-world evidence for HTA bodies/payers decision making
Source: Int J Technol Assess Health Care. 2025 Jun 17;41(1):e52. doi: 10.1017/S0266462325100238 (PMC12350084; doi:10.1017/S0266462325100238)
Supplement: Jaksa et al. supplementary material [file S0266462325100238sup001.docx]

**Stakeholder Actions to Generate Better RWE for HTA/Payer Decisions**

**Comparison: 2020 vs 2025 versions**

| **Stakeholder Actions – 2020 version** | **Stakeholder Actions – 2025 version** | | |
| --- | --- | --- | --- |
| **Payers and HTA bodies – Payer/HTA Collaboratives** | |  |  |
| No correspondent | 2.1 Use HTA/Payer collaboratives (such as Joint Nordic HTA-Bodies, the Beneluxa initiative etc) to encourage and enhance joint work on use of RWD in initial access decisions, managed entry agreements and re-assessments.  2.2 Develop a joint voice on RWD issues to feed into policy and system developments such as the European Health Data Space (EHDS) and the Data Analysis and Real-World Interrogation Network (DARWIN-EU) and the implementation of the HTA Regulation, with clear accountability for this task potentially assigned to an entity such as NCAPR.  2.4 Work with Regulators to understand and influence their international activities to develop harmonized methods and guidance for RWD collection. In Europe, incorporate RWD/E needs and guidance in the implementation of the HTA Regulation through Joint Scientific Consultations and Joint Clinical Assessments. | | |
| 1.1 Collaborate with academia to better understand the potential of new statistical, econometric, and modeling approaches to develop robust RWE for use in Payer/HTA decisions. | 2.3 Co-create joint learning platforms with other decision-makers and stakeholders in the RWD/E community.  2.7 Collaborate with companies, clinical teams, academia and other stakeholders on study protocols, study governance, analyses, and reporting to encourage a common understanding of HTA requirements and to promote open access to documents and findings. | | |
| 1.2 Encourage industry to engage in multi-stakeholder dialogues to discuss evidence generation plans including RWD collection. | 2.3 Co-create joint learning platforms with other decision-makers and stakeholders in the RWD/E community.  2.5 Collaborate to expand Early Dialogue/scientific advice/Joint Scientific Consultation to cover RWE generation throughout the product life cycle to address uncertainties in clinical and cost effectiveness. Recognise the need for agile processes to adapt to evolving knowledge, treatment options and RWD and RWE requirements.  2.6 Encourage and support industry to consider possibilities to expand post-authorisation studies and data collection plans intended to support regulatory decision making to also address HTA/Payer evidence needs.  2.7 Collaborate with companies, clinical teams, academia and other stakeholders on study protocols, study governance, analyses, and reporting to encourage a common understanding of HTA requirements and to promote open access to documents and findings. | |  |
| 1.3 Use joint processes (multi-HTA and with regulators) to document evidence gaps and key uncertainties in the clinical (and economic) evidence and identify which areas might be addressed by patient-relevant RWE. | 2.8 Collaborate with regulators on common frameworks for data quality assessment, data standardisation efforts and methodologies for feasibility assessment. Advise health data holders of the common requirements so that they can develop their datasets accordingly. | |  |
| 1.4 Document the regulatory post-licensing evidence generation (PLEG) obligations and the additional Payer/HTA PLEG needs. Establish what PLEG is needed at national, regional, and European levels. | 2.11 Compile available documents (protocols and reports) describing examples of post launch RWD collection and RWE generation required by HTA/payers, utilizing an existing portal if available. | |  |
| 1.5 For individual HTAs, agree the core data set that is required for HTA reappraisal, within a reasonable timeframe, so that common data collection protocols can be agreed across countries and joint analyses performed. |  | |  |
| 1.6 Engage with the clinical community, particularly European Reference Networks (ERNs) to avoid conflicting or duplicative data collection. |  | |  |
| 1.7 Develop method guides to show how RWE will be critically assessed and how the validity and applicability of RWD/RWE from another country/health setting will be determined. | 2.7 Collaborate with companies, clinical teams, academia and other stakeholders on study protocols, study governance, analyses, and reporting to encourage a common understanding of HTA requirements and to promote open access to documents and findings.  2.9 Pilot the use of existing RWD/E methods guidance and tools in assessments. Share feedback with guidance authors and HTA/Payer community.  2.10 After piloting existing guidance, develop harmonized RWE guidance jointly with academia and industry, particularly to support implementation of the HTA Regulation. Review the guidance regularly to take account of new methodological developments and experience of use (living guidance). | |  |
| 1.8 Encourage the development of a public portal registering RWE studies that may be used in decision making and when fully established only accept studies previously registered and reported on the portal. | 2.11 Compile available documents (protocols and reports) describing examples of post launch RWD collection and RWE generation required by HTA/payers, utilizing an existing portal if available. | |  |
| **Payers and HTA bodies – National Payers/HTA Bodies** | |  |  |
| No correspondent | 1.1 Create a shared vision that conceptualizes a future state for the use of real-world data (RWD) in health technology assessment (HTA)/Payer processes. Ensure that clear roles and responsibilities are designated for oversight and achievement of the shared vision.  1.7 Publish examples where RWE has influenced pricing and reimbursement decisions or reassessments. Also share case studies that identify methodological areas requiring development. | |  |
| 2.1 Encourage establishment of national processes for data collection, analysis, critical assessment, and appraisal that enable collection of relevant and reliable RWD to inform access and reimbursement decisions and optimize use of highly innovative technologies to achieve the best outcomes for patients. |  | |  |
| 2.2 Inform national stakeholders about RWE needs for HTA and engage in developments to enable secondary use of health and social care data in the national context. | 1.5 Influence national developments on the secondary use of health data. Communicate HTA/Payer needs regarding, for example, types of data, data linkage and data quality, ensuring these needs are considered and integrated into national governance frameworks. | |  |
| 2.3 Seek to harmonize requests to Marketing Authorization Holders for additional RWD collection with other HTA bodies and the regulator. | 1.3 Collaborate with other HTA bodies and Payers (e.g. via networks such as the National Competent Authorities for Pricing and Reimbursement (NCAPR) or the Medicine Evaluation Committee (MEDEV)) and regulatory authorities to align post-launch evidence generation (PLEG) requirements needing national data collection. Focus on critical endpoints and requirements for data quality. | |  |
| 2.4 Engage with stakeholders to agree responsibility for the conduct and financing of RWD collection and analysis. |  | |  |
| 2.5 Engage with patient organizations to ensure data is collected on those outcomes that matter most to patients. |  | |  |
| 2.6 Build capacity in data analytics and critical assessment of RWE studies. | 1.2 Overcome fragmentation and lack of collaboration between HTA bodies and payers by implementing the necessary infrastructures, aligning processes, and upskilling competencies for effectively requesting, producing, and utilizing real-world evidence. | |  |
| 2.7 Actively explore the opportunity to use RWD from other countries/across country (e.g., European registries), preferably in collaboration with other HTA bodies, in consultation with industry, taking account of national/context specific limitations of the data. |  | |  |
| 2.8 Request RWE generation plans from companies, including protocols or plans for data collection and analysis of RWE studies related to HTA. | 1.4 Initiate and engage in multistakeholder discussions with companies about RWE generation plans. Determine which data are transferable and identify additional local RWD that may be needed to inform decision-making, aligning with other jurisdictions when possible. | |  |
| 2.9 During assessment, consider the feasibility of RWD collection, possibly combined with an OB-MEA (nationally or in collaboration with other countries) to manage important uncertainties and enable reassessment. | 1.6 Require that feasibility assessments, study protocols, details of data extractions and study reports are made publicly available. | |  |
| 2.10 When an OB-MEA is used, engage with patients and clinicians to plan for potential routine adoption or modification of reimbursement conditions or disinvestment at the end of the agreement. |  |  |  |
| **Regulators** | |  |  |
| 3.1 Promote use of parallel multi-HTA Scientific Advice/Early Dialogue processes including all relevant stakeholders at various points in the development of a highly innovative technology. | No actions for regulators in the new stakeholder actions. | |  |
| 3.2 Develop, in co-creation with all stakeholders, guidance on generic RWE issues discussed in Scientific Advice/Early Dialogues for highly innovative technologies. |  |  |  |
| 3.3 Continue to support methodological discussions with industry about non-RCT methodologies through methods qualification and multi-stakeholder scientific advice. |  |  |  |
| 3.4 Involve HTA bodies in initiatives related to improving the quality of RWD such as registry qualification, quality standards for big data/RWD etc. |  |  |  |
| 3.5 For highly innovative technologies with conditional marketing authorization (MA) or MA under exceptional circumstances, involve HTA bodies in discussions about requirements for post-marketing data collection. |  |  |  |
| **Pharmaceutical industry** | |  |  |
| No correspondent | 3.3 Work together across company functions and national affiliates involved in RWE generation and analysis to develop a common understanding of national/regional HTA/Payers needs for RWE.  3.4 Lead discussions about transportability of real-world data (RWD) across borders and support efforts to align data collection requirements across jurisdictions.  3.5 Continue to drive discussions about use of, and alignment of, Outcomes-Based Managed Entry Agreements (OBMEA)/Post-Launch Evidence Generation (PLEG).  3.9 Engage and support operationalisation of the HTA Regulation to highlight need for RWE in the first two tranches of JCAs and encourage development of clear guidance about assessment of RWE in the EU HTA context. | |  |
| 4.1 Create a RWE generation plan early in development, which addresses essential data elements for HTA not covered in the clinical trial program and that might be studied in a real-world setting. | 3.1 Discuss plans for real-world evidence (RWE) generation as part of industry (integrated) evidence plans at scientific advice meetings. Do this at several points during the lifecycle of the medicine, to develop understanding of what RWE might be valuable to HTA/Payers as clinical evidence and knowledge about the technology/disease evolves. | |  |
| 4.2 Discuss the RWE generation plan at various stages throughout the technology life cycle including regulators, payers, HTA bodies, clinicians, and patients whenever possible. |  |  |  |
| 4.3 Ensure the study (protocol) and statistical analysis plans for RWE studies that are answering major HTA questions are available to HTA bodies to provide transparency about the methods used to obtain and analyze data. | 3.2 Ensure transparency around the design, conduct, and analysis of RWE studies that are agreed to be pivotal to health technology assessment (HTA)/Payer decision making, e.g. using published tools to document data capture, management and analysis, following RWE guidance/frameworks. | |  |
| 4.4 Support the development of a public portal that provides details about the design and results of major RWE studies (ala RCT registries), particularly hypothesis evaluating treatment effect studies. | 3.10 Share, publish and enable discussion of case studies to show how RWE has been assessed and used in HTA/Payer decision-making (in assessment and post-launch), focussing on specific issues (e.g. external control arms, transportability), and challenging cases scenarios (e.g. rare disease) etc. | |  |
| 4.5 Drive non-competitive, multi-company, and multi-stakeholder collaborations about the development of robust RWE for diseases treated by highly innovative technologies. | 3.7 Collaborate across companies (e.g. in a specific disease area or for a class of medicines) to agree on synergies in methods and processes for RWE in that specific case (e.g. core datasets, analytical approaches, etc). | |  |
| 4.6 Enact recommendations from the ISPOR RWE Transparency Partnership Initiative. |  | |  |
| 4.7 Use reliable data collection methods for RWD, including eHealth and digital tools and develop best practices as digital approaches evolve. | 3.6 Explore use and analysis of digital apps to capture patient-relevant outcomes, particularly to inform OBMEA.  3.8 At an early stage of product development, engage with clinical networks to create or further develop registries and databases following unified data standards, to collect high quality and interoperable data. | |  |
| **Registry-holders / Disease registry holders** | |  |  |
| No correspondent | 6.4 Share and publish case studies of where real-world data (RWD) from disease registries has been used in to support HTA/Payer decision making. | |  |
| 5.1 ERNs should continue to be developed and encouraged to engage with decision makers to discuss registries and other sources of RWD. |  | |  |
| 5.2 Collaborate with regulatory authorities, HTA bodies and industry at an early stage in the development of a highly innovative technology to identify if existing registries could be used to resolve uncertainties during the development phase or for post-launch data collection, or discuss potential for new registries. | 6.2 Form multi-stakeholder partnerships to support use of data for HTA/payer purposes, including public/private partnerships.  6.3 Engage with HTA/Payers and industry to explain the construct and purposes of disease registries, discuss their potential and limitations, and agree with HTA/Payers how disease registries and RWE studies will be assessed, building on existing tools (e.g. tools to report registry quality overall, and fitness-for-purpose evaluations for individual RWE studies). | |  |
| 5.3 Review quality standards for registries issued by EMA and EUnetHTA and apply them. |  |  |  |
| 5.4 Collaborate on an ongoing basis with HTA bodies/regulatory authorities/manufacturers to determine if a standard set of data can be collected and shared to enable pooling with other data sources for analysis. | 6.1 Participate in multi-stakeholder dialogues about real-world evidence (RWE) generation for a specific medicine, or group of medicines, to discuss the potential for a disease registry to be used for regulatory and health technology assessment (HTA)/Payer purposes and ensure realism about data quality (availability for main outcomes and trade-offs for different data capture algorithms). | |  |
| 5.5. Collaborate with European and national policy makers to develop governance structures that facilitate data quality and accessibility. | 6.5 Develop governance processes that enable sharing of disease registry data and linkage to other data sources (e.g. administrative data) for HTA/Payer purposes (including  patient consent mechanisms, protocols, data management etc).  6.6 Align to RWD standards to ensure data meets quality standards required by HTA bodies. | |  |
| 5.6 Create incentives to build and maintain disease-based, rather than product-specific, registries. |  | |  |
| 5.7 All Registries should follow the FAIR (Findable, Accessible, Interoperable, Re-usable) data principles and provide “privacy by design”—ensuring data protection through technology design. | 6.5 Develop governance processes that enable sharing of disease registry data and linkage to other data sources (e.g. administrative data) for HTA/Payer purposes (including  patient consent mechanisms, protocols, data management etc). | |  |
| 5.8 If available in the health system, registries should use the unique patient-id to enable linkage with other health data sources. |  |  |  |
| **Clinicians** | **Clinical teams** | |  |
| No correspondent | 4.1 EU clinical networks should engage in developments related to the European Health Data Space to support public and political awareness of the value of secondary use of health data. | |  |
| 6.1. ERNs and other clinical networks should systematically collaborate with regulators and Payer/HTAs when establishing their registry or other form of RWD collection to ensure it is fit for all purposes including HTA. | 4.2 EU clinical networks and clinical trials collaborative groups (such as European Reference Networks, the EU cancer mission networks, the European Organisation for Research and Treatment of Cancer and other disease specific study groups) should systematically involve patients to collect patient relevant outcomes including nutritional status and co-morbidities, and collaborate with regulators and Payer/health technology assessment (HTA) bodies to ensure data collection systems are fit for all purposes, including HTA requirements pre and post launch. | |  |
| 6.2 Enhance the H2020 EU Joint-Program for rare diseases to include regulators and HTA bodies in order to better understand their needs. |  | |  |
| 6.3 ERNs and other clinical networks should advise on the most suitable and efficient way for health systems to collect RWD. | 4.4 Clinical networks and study groups should advise on the most suitable and efficient way for health systems to collect real-world data (RWD) to avoid multiplicity of data entry and clarify the support clinical teams require to collect good quality RWD.  4.5 Clinical networks and clinical trials collaborative groups should encourage health systems to involve clinical teams and patients in the design of data collection systems and associated governance structures to ensure processes are efficient and clarify the support clinical teams require to collect good quality real world data. | |  |
| 6.4 Encourage clinical communities to collect high quality health data (administrative, registry, audit etc.) that is required for Payer/HTA decisions. | 4.3 Medical faculties should include educational programmes on the value of health system data to improve delivery of care, patient outcomes and inform health system decision making, such as HTA evaluations of new healthcare interventions. | |  |
| 6.5 Promote the use of data-driven shared decision-making processes for optimization of treatment of the individual and care pathways. | 4.6 Clinicians should seek to ensure that local information governance systems are designed to deliver optimal care for patients (across providers and departments), and to encourage informed consent processes based on unified ethical principles that are intelligible to patients, in all required languages. | |  |
| 6.6 Understand the importance and relevance of the role of RWD in OB-MEA in order to align on right usage and adherence to collect the data needed. |  | |  |
| **Patient Groups** | | |  |
| 7.1 Develop patient group expertise and capacity to be co-creators of RWE to ensure a holistic, patient-centered approach. | 5.1 Ensure that opportunities and resources to develop patient expertise in the field of RWD are clearly communicated to the patient community to develop skills that support multi-stakeholder and patient-centred generation of real-world evidence (RWE) to inform health technology assessment (HTA) and to engage in policy and system developments relating to use of health data. | |  |
| 7.2 Develop EU or international patient group collaborations to engage in RWD initiatives and ensure that outcomes that matter to patients are collected. | 5.2 International patient groups should continue to engage in real-world data (RWD) initiatives, such as Innovative Health Initiatve (IHI) projects, regulatory and HTA led activities, and policy developments such as the European Health Data Space (EHDS). | |  |
| 7.3 Support the development of patient information and informed consent for RWE studies, clearly explaining study expectations (e.g., regularity of clinic visits and assessments) and treatment discontinuation rules. | 5.5 Support the development of efficient informed consent processes for secondary use of health data. This will necessitate a rethinking of the type of consent model in use from ‘broad to ‘dynamic’ to encourage accelerated and increased patient participation. | |  |
| 7.4 Disseminate clear unbiased, patient-relevant information about RWD and RWE to patient communities, including the value of secondary use of data. | 5.6 Disseminate clear, unbiased, patient-relevant information about RWD and RWE to patient communities, including the value of secondary use of data and information to support Post-Launch Evidence Generation. | |  |
| 7.5 Provide recommendations on novel and efficient collection methods for RWD (e.g., devices, wearables, and mHealth). | 5.7 Engage with clinicians, academics and decision-makers to discuss how patient-relevant data from novel collection methods (such as wearables, apps etc) can be used in decision-making, to help build a predictable pathway for use of these novel RWD collection approaches. | |  |
| 7.6 Help promote the scientific and policy value of data collection and provide access within strict governance frameworks ensuring appropriate confidentiality, e.g., via the #DataSavesLives initiative. | 5.3 Seek to influence the implementation of the EHDS and understand its implications for national data collection systems, in particular to ensure that patients have access to their own data. | |  |
| 7.7 Work with stakeholders to encourage alignment of views on identification, collection, analysis, and evaluation of RWD for decision making. | 5.4 Support development of a process for iterative multi-stakeholder dialogues throughout the lifecycle of a medicine to encourage alignment of views on identification, collection, analysis and evaluation of RWD for decision-making.  5.8 Patient Groups who might also be disease registry holders or have developed disease specific data collection approaches, should agree with stakeholders how to integrate such evidence with other data for highly innovative medicines. | |  |
| **RWD/Analytics Groups** | | |  |
| No correspondent | 7.5 Raise awareness of differences between HTA body RWE frameworks and how these differences relate to differences in HTA body remits and RWE needs.  Where appropriate, engage in dialogue with HTA bodies to encourage harmonization of fit-for-purpose methods. | |  |
| 8.1 Engage with HTA bodies and other stakeholders in demonstration projects related to highly innovative technologies. | 7.4 Work with HTA bodies to share and operationalize the implications from available demonstration projects (e.g., emulation studies, newly developed methods, tools, repositories etc) to build mutual understanding and trust in RWE; explore which are most helpful or need adaptation and support collaboration to create a harmonized RWE toolkit. | |  |
| 8.2 Collaborate with industry and HTA bodies to generate RWE for OB-MEA. |  | |  |
| 8.3 Contribute to research collaborations that lead to published guidance on the use of RWD for specific research questions relevant to HTA of highly innovative technologies. | 7.1 Support development of a repository of empirical evidence/case law about what real-world evidence (RWE) was fit for purpose in health technology assessment (HTA) and what was not, as well as RWE case studies that highlight data quality and methodology examples.  7.2 Continue to operationalise and share/publish methodologies that address known HTA/Payers’ concerns with use of RWE through HTA-friendly tools, demonstration projects, case studies etc.  7.3 Build RWE analytics knowledge base and support RWE assessment and generation within HTA bodies linking to new policy initiatives such as the HTA Regulation.  7.7 Create a standard world-wide library of definitions for key aspects covering diagnoses, outcomes, covariates etc and algorithms that have been validated. | |  |
| 8.4 Work with stakeholders to develop reporting standards, including full transparency, software audit trails, and study pre-registration. | 7.4 Work with HTA bodies to share and operationalize the implications from available demonstration projects (e.g., emulation studies, newly developed methods, tools, repositories etc) to build mutual understanding and trust in RWE; explore which are most helpful or need adaptation and support collaboration to create a harmonized RWE toolkit.  7.6 Where there is substantial evidence and agreement across stakeholders on use cases, standards, and analytical methods and where articulating guidance would benefit researchers, support HTA bodies in developing  detailed published guidance. | |  |
| 8.5 Collaborate with industry, regulators, and HTA bodies to enhance the efficiency of preparation and submission of RWE that facilitates transparency, addresses proprietary data, and privacy challenges and includes robust data governance. |  |  |  |
| 8.6 Work with stakeholders to develop criteria to evaluate RWD’s fitness for purpose, provenance, transparency, and adherence to governance standards in order to inform the evaluation of RWE. | 7.7 Create a standard world-wide library of definitions for key aspects covering diagnoses, outcomes, covariates etc and algorithms that have been validated.  7.8 Work with industry to ensure they are following published standards/best practices for RWE generation (e.g., transparency, data quality, etc).  7.9 Work with data custodians to explain the requirements of decision-makers to clearly demonstrate data quality and encourage standardised documentation they can use for all RWE studies. | |  |
| 8.7 Publish analytical methods to generate reliable RWE for HTA. |  |  |  |
